# Engineering growth factor gradients to drive spatiotemporal tissue patterning in organ-on-a-chip systems

**DOI:** 10.1177/20417314251326256

**Published:** 2025-04-18

**Authors:** Timothy Hopkins, Swati Midha, Simon Grossemy, Hazel R. C. Screen, Angus K. T. Wann, Martin M. Knight

**Affiliations:** 1 Centre for Predictive In Vitro Models, Queen Mary University of London, UK; 2 Centre for Bioengineering, School of Engineering and Materials Science, Queen Mary University of London, UK; 3 School of Biological Sciences, Institute for Life Sciences, University of Southampton, UK; 4 Kennedy Institute of Rheumatology, Nuffield Department of Orthopaedics, Rheumatology and Musculoskeletal Sciences, University of Oxford, UK

**Keywords:** organ-on-a-chip, spatial patterning, growth factors, morphogens, gradient, endochondral ossification

## Abstract

Spatial heterogeneity plays a key role in the development and function of human tissues and therefore needs to be incorporated within in vitro models to maximise physiological relevance and predictive power. Here, we developed and optimised methods to generate spatial heterogeneity of hydrogel-embedded bioactive signalling molecules within organ-on-a-chip (OOAC) systems, to drive spatiotemporal tissue patterning through controlled stem cell differentiation. As an exemplar application, we spatially patterned bone morphogenetic protein-2 (BMP-2) in both closed-channel and open-chamber OOAC formats. The resulting BMP-2 gradient in 3D heparin methacryloyl/gelatin methacryloyl, successfully drove spatially divergent differentiation of human bone marrow-derived stem cells into bone-like and cartilage-like regions, mimicking the process of endochondral ossification in the growth plate. The application of hydrogel-embedded morphogens to drive spatial tissue patterning within OOAC systems represents a significant technological advancement and has broad-ranging applicability for a diverse range of tissues and organs, and a wide variety of OOAC platforms.

## Introduction

Spatial heterogeneity is evident within a wide range of human tissues and organs, with gradients and interfaces playing a crucial role in development, function and dysfunction.^1,2^ This spatial tissue patterning is frequently driven by variation in soluble, and extracellular matrix (ECM)-bound, morphogens, such as growth factors.^3^ Morphogen gradients drive the formation of cellular gradients through the spatiotemporal regulation of local cell proliferation, migration and differentiation.^4,5^ In turn, cellular gradients define extracellular gradients, including transitions in ECM composition, architecture and mechanical properties, which reciprocally influence cell behaviour.^4,6^ Thus, morphogen gradients are pivotal in the regulation of normal human tissue physiology. In addition, the disruption of morphogen gradients has pathological implications, contributing to many human congenital disorders including chondrodysplasias^7^ and neurodevelopmental disorders.^8^

Hence, the incorporation of morphogen gradients into the next generation of in vitro models, will provide more accurate, physiologically relevant representation of the complexity of intra- and inter-tissue heterogeneity, in health and disease, and therefore better prediction of human biology and response to therapeutics.

Organ-on-a-chip (OOAC) technology combines biology and engineering to offer improved, dynamic, in vitro models of tissue function that more accurately replicate in vivo physiological responses compared to simplistic in vitro models.^9^ These models also have the advantage of reducing reliance on poorly predictive in vivo animal models and support the drive to reduce the overall use of animals in science. Accurate reflection of dynamic in vivo function in OOAC is achieved through the incorporation of key human cell types and components of the extracellular microenvironment that regulate cellular phenotype and function. These components include: tissue-specific ECM composition, tissue organisation, tissue stiffness, mechanical stimulation and interaction with adjacent tissues such as with the vasculature which facilitates nutrient supply, drug delivery and waste removal.^10^ Spatial patterning of morphogens has been successfully demonstrated in more conventional, 3D, hydrogel-based, in vitro models using a variety of methods, including surface immobilisation,^3^ microsphere delivery,^11^ diffusion,^12^ 3D printing^13^ and buoyancy-driven techniques.^4^ However, many of these techniques are currently unsuitable, and require significant optimisation, for use within OOAC due to issues associated with accessibility, scalability, size limitations, microfluidic boundary conditions, 3D spatial control, in situ cross linking, biological compatibility and sterility. The current dearth of spatial patterning techniques suitable for OOAC models represents a significant roadblock in the delivery and translation of these models.

There have been previous successful attempts to generate functionally relevant gradients of soluble factors within microfluidic, OOAC systems, for example of pro-angiogenic factors^14^ and oxygen.^15^ While these elegant solutions have proved useful for specific applications, the establishment and maintenance of gradients in these cases was reliant on the constant flow of media containing the factor of interest. Thus, their ease of use and broader applicability to other OOAC platforms is limited. The aim of the present study was to optimise and validate methods for spatial patterning that could be applicable to as broad a range of OOAC systems as possible. The majority of these systems can be broadly stratified into ‘closed-channel’ (e.g. Emulate Chip-S1^®^, BioMimiX^®^ uBeat^®^, MIMETAS Organoplate^®^) or ‘open-chamber’ (e.g. Emulate Chip-A1^®^, Tissuse GmbH Humimic, CN-Bio PhysioMimix ^®^, MIMETAS Organograft^®^) formats. Further subdivisions can be found within these categories. For example, of the ‘closed-channel’ chips, there are those in which compartments are separated by a porous membrane (e.g. Emulate Chip-S1^®^) and other in which they are separated by pillars or Phaseguides™ (e.g. MIMETAS Organoplate^®^).

Therefore, we sought to generate spatial heterogeneity in multiple representative OOAC formats: (1) closed-channel with membrane, (2) closed-channel with phase-guides™ and (3) open-chamber OOAC, using the Chip-S1^®^ (Emulate Inc., Boston, USA), Organoplate^®^ (MIMETAS, Oegstgeest, Netherlands) and Chip-A1^®^ (Emulate Inc.) as respective, representative examples of each of these formats.

As an exemplar demonstration of the capability of the optimised methodology, we developed spatial patterning of the growth factor bone morphogenetic protein-2 (BMP-2), within OOAC, in order to drive osteogenic and chondrogenic differentiation of human bone marrow derived stem cells. The osteochondral junction is one of the archetypal transitional interfaces in the human body and is the phased border between cartilage and bone, as found in human joints.^16,17^ The osteochondral interface develops at the growth plate by a process called endochondral ossification, whereby gradients of morphogens, including BMP-2, influence the behaviour of the resident stem cell population, driving spatially diverse differentiation.^18^

In the present study, we demonstrate successful patterning of BMP-2 gradients in three distinct OOAC formats, and show how this generated spatial tissue patterning of bone-like and cartilage-like regions within the OOAC.

## Materials and methods

### Organ-on-a-chip configurations

**Figure 1.**
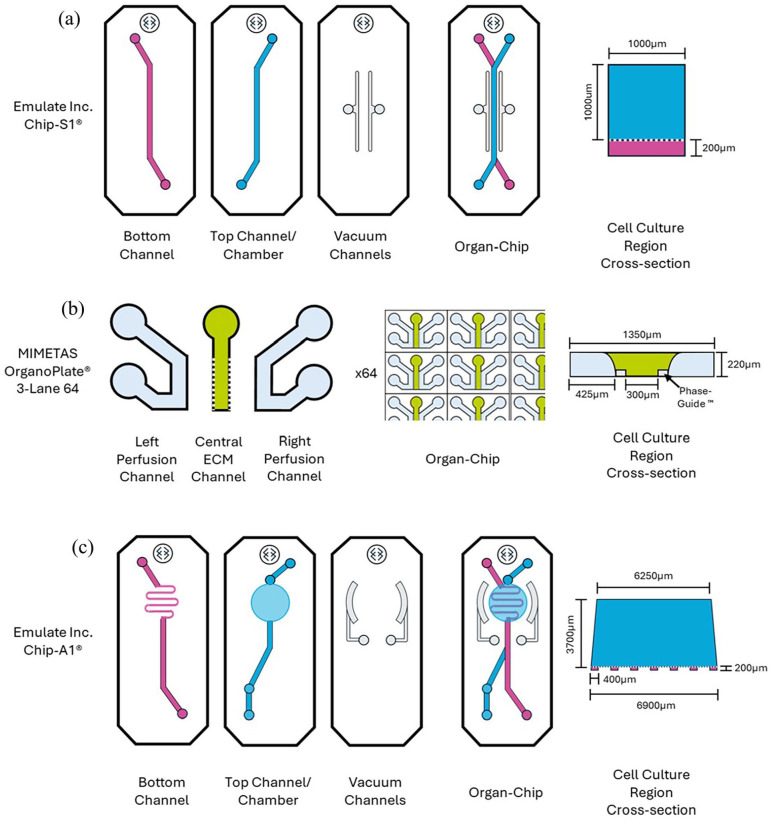
Configuration of (a) Chip-S1^®^ (Emulate Inc.), (b) Organoplate^®^3-lane 64 (MIMETAS) and (c) Chip-A1^®^ (Emulate Inc.). See text for detailed description of each configuration. A cross-section of the coculture region for each of the chip formats is shown (not to scale).

**(1) Closed-channel with membrane format:** The Chip-S1^®^ (Emulate Inc.; Figure 1(a)) is a PDMS-based OOAC comprising two overlapping microfluidic channels: a top channel (1 mm width × 1 mm height) and a bottom channel (1 mm width × 0.2 mm height), separated by a by a 50 µm thick, porous membrane (7 µm pore diameter). The channels are accessed via the ports at either end of each channel, termed the inlets and outlets. Each channel is connected by microfluidics to its own medium reservoir using the Pod™ system (Emulate Inc.) such that each can be supplied individually with required media formulations, which flows unidirectionally from the inlets to the outlets. When the top channel is loaded with a hydrogel, there is no media flow in this channel with media exchange coming solely from the bottom channel. Two parallel vacuum channels either side of the culture channels and can be used to apply uniaxial cyclic tensile strain across the membrane.
**(2) Closed-channel with Phaseguides**™ **format**: The OrganoPlate^®^ 3-lane 64 (MIMETAS; Figure 1(b)) is a 384-well tissue culture plate-based OOAC platform with a glass bottom, supporting 64 independent chips on a single plate. Each chip comprises three adjacent microfluidic channels: a central ECM channel (300 µm width × 220 µm height) with perfusion channels on either side (425 µm width × 220 µm height), separated by Phaseguides™ (100 µm width × 50 µm height) allowing diffusion of media and barrier-free, cell-cell interactions. The channels can be accessed via dedicated inlets and outlets consisting of small holes for chip loading, except the central ECM channel, which comprises of an inlet only. Each microfluidic channel has its own media reservoir which can be accessed from the top, like a traditional cell culture plate. During cell culture, the OrganoPlate^®^ is placed on a rocker (OrganoFlow^®^) and titled between +14° and −14° every 8 min to allow gravity-driven, bi-directional flow through the perfusion channels. Each chip consists of an observation window for in-situ imaging.
**(3) Open-chamber format:** The Chip-A1^®^ (Emulate Inc.; Figure 1(c)) is a PDMS-based OOAC comprising a bottom channel (0.4 mm width × 0.2 mm height) that winds back and forth beneath an accessible, open culture chamber (a shallow, truncated cone in shape, 6.9 mm diameter at the base reducing to 6.25 mm at the top; height 3.7 mm, 125 µl volume). The top culture chamber and bottom channel are separated by a 50 µm thick, porous membrane (7 µm pore diameter). The bottom channel can only be accessed via the inlet and outlet, but the culture chamber can be accessed from the top, analogous to a traditional cell culture plate and sealed with a hinged lid. Again, each compartment is connected to its own medium reservoir by microfluidics using the Pod™ system which enables the individual supply of media to each, with flow from the inlets to the outlets. When the culture chamber is loaded with a hydrogel, media in this compartment flows over the top of the gel. Two, curved vacuum channels either side of the culture chamber can used to apply uniaxial cyclic tensile strain across the membrane.

Prior to gel seeding in either Emulate Inc. chip format, the inner surfaces of the channels/chambers were activated according to standard protocols (www.emulatebio.com; ‘Basic Research Kit Protocol: EP223’). In brief, 0.5 mg/ml ER-1 solution (proprietary, Emulate Inc.) was added to each channel/chamber and the chips were then treated with UV light for 10 min. The channels were aspirated, and the process repeated with additional ER-1 solution. Channels were then washed with ER-2 solution (proprietary, Emulate Inc.) followed by phosphate buffered saline (PBS; Gibco). The PBS was aspirated, and the OOAC were placed in a glove bag supplied with nitrogen for at least 2 h prior to gel seeding to deplete oxygen, which was critical for the curing of hydrogels within the OOAC.

### Hydrogel formulation

#### Materials synthesis and characterisation

Gelatin methacryloyl (GelMA) synthesis was performed as previously described^1,19^ with slight modifications. Briefly, 5 ml of methacrylic acid (MAA, Sigma-Aldrich, UK) was reacted with 10% w/v of gelatin (Gelatin from porcine skin, type A, gel strength 300 bloom, Sigma-Aldrich, UK) dissolved in deionised water under controlled parameters (55°C temperature, 500 rpm speed) while minimising air exposure to facilitate methacryloyl substitution. After 3 h, unreacted MAA was removed by centrifugation and the GelMA was dialysed, freeze-dried and stored at −20°C.

Heparin methacryloyl (HepMA) synthesis was performed as before.^1^ Briefly, fivefold excess of MAA was added to a 2% w/v solution of heparin (sodium salt from hog intestine, Tokyo Chemical Industry, UK) dissolved in deionised water and stirred overnight, with pH adjusted to 8.5 periodically. After 24 h, the resultant solution was precipitated in ethanol, rehydrated and dialysed against deionised water. Purified HepMA was freeze-dried and stored at −20°C.

To quantify the methacryloyl substitution of unmodified gelatin, GelMA, unmodified heparin and HepMA, 10 mg of each of the freeze dried macromers were dissolved separately in 800 µl deuterium oxide containing 0.05% 3-(trimethylsilyl) propionic-2,2,3,3-d4 acid (TMSP) as an internal standard and the spectra obtained using a Bruker 400 MHz, 1H-NMR spectrometer (Bruker, Billerica, USA). Rheological measurement of GelMA was conducted using a Discovery Hybrid Rheometer (DHR-3; TA Instruments, Denver, USA) equipped with a 20 mm parallel top plate and a TA UV-light accessory. Samples were placed between the top plate and the bottom window (20 mm diameter), maintaining a gap of 250 μm. Rheological measurement was initiated prior to UV radiation to capture gelation dynamics and hydrogel moduli. UV irradiation was administered using an Omnicure^®^ S2000 UV light directly at the start of the measurement oscillations, for 400 s. The specific irradiance intensity utilised was 17 mW/cm^2^, matching the intensity of the Emulate Inc. UV boxes used with the OOAC. Oscillations were induced at a fixed strain of 1% and a frequency of 1 Hz to ensure measurements remained within the linear viscoelastic region of the samples obtained from the amplitude sweep. In situ time sweeps were conducted at a controlled temperature of 37°C, employing a strain of 1% and a frequency of 1 Hz.

**Figure 2.**
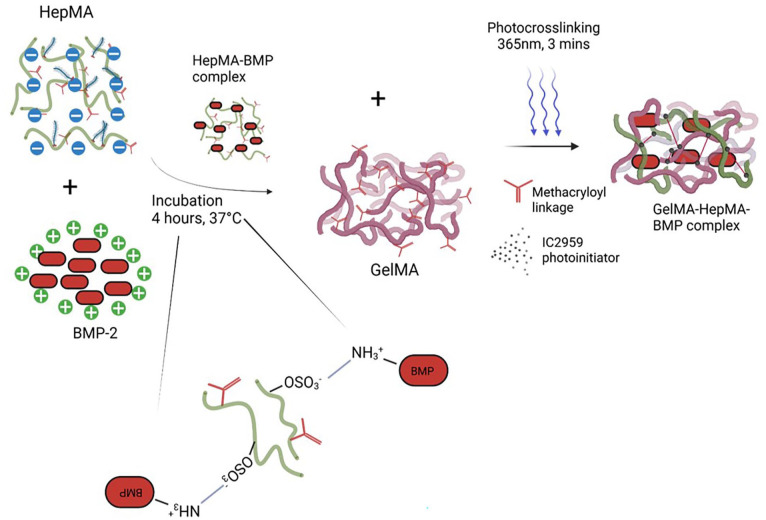
Schematic overview of the sequestration of BMP-2 by HepMA, the addition of this complex to GelMA and its immobilisation by UV curing in the presence of the photoinitiator.

Figure 2 depicts the process by which BMP-2 is sequestered by HepMA, the addition of this complex to GelMA and its subsequent photo crosslinking.

#### Hydrogel formation

Irgacure 2959 (2-hydroxy-4′(2-hydroxyethoxy)-2-methylpropiophenone; Sigma-Aldrich) was used as the photoinitiator. A 1 mg/ml solution of Irgacure 2959 (0.1% w/v) was prepared in PBS, and GelMA was rehydrated with the stock Irgacure 2959 solution, to a final concentration of 10% (w/v), overnight at 4°C before being transferred to 37°C for a few hours before mixing with cells. HepMA was employed as a macromolecular host, to sequester cationic proteins such as growth factors, that could be covalently crosslinked to itself and to the surrounding GelMA.^1^ A 1 mg/ml stock of HepMA was prepared in PBS.

Bone morphogenetic protein-2 (BMP-2) was utilised as a representative example of a growth factor that could be sequestered by HepMA, and that could therefore be inhomogenously distributed throughout the GelMA by patterning the HepMA. For visualisation and quantification of BMP-2 patterning, a 200 µg/ml stock solution of fluorescein isothiocyanate (FITC)-conjugated BMP-2 (Generon, Maidenhead, UK) was prepared in sterile 4 mM HCl. HepMA and BMP-2 (final concentration 12.5 µg/ml) were mixed for at least 4 h prior to gel fabrication to ensure equilibration (Figure 2). For the buoyancy method utilised in the Chip-A1^®^, 5% (w/v) Ficoll-400 (Sigma-Aldrich, Dorset, UK) was used as a density modifier. Final gels were formulated, as detailed below, and stored at 37°C until use.

Closed-channel formats (with membrane (Chip-S1^®^) and with Phaseguides™ (OrganoPlate^®^)):

1. Gel 1: 10% (w/v) GelMA, 1 mg/ml Irgacure 2959.
2. Gel 2: 10% (w/v) GelMA, 1 mg/ml Irgacure 2959, 10 µg/ml HepMA, 12.5 µg/ml BMP-2.

Open-chamber format – Chip-A1^®^:

1. Base gel: 10% (w/v) GelMA, 1 mg/ml Irgacure 2959, 10 µg/ml HepMA, 12.5 µg/ml BMP-2, 5% (w/v) Ficoll-400.
2. Injection gel: 10% (w/v) GelMA, 1 mg/ml Irgacure 2959.

### Spatial patterning in OOAC

Following the activation of the OOAC, all subsequent steps were performed in a nitrogen-filled glove bag to maintain the oxygen depletion necessary for curing the gels within the chips.

**Figure 3.**
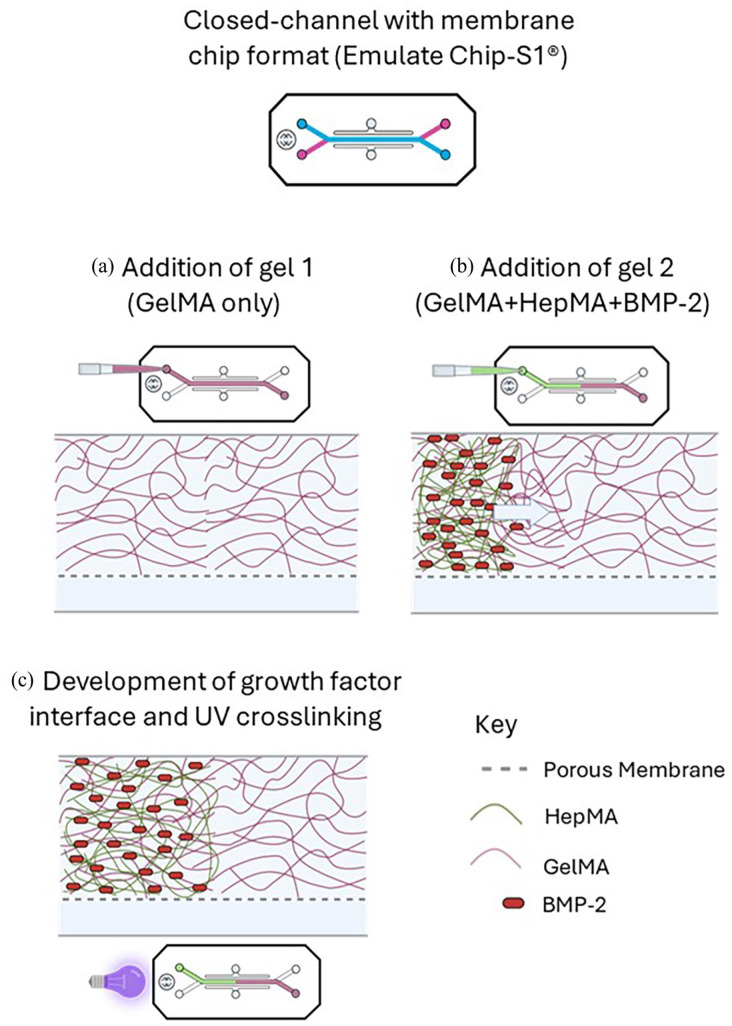
Method to generate spatial distribution of BMP-2 in Emulate Chip-S1^®^: (a) addition of Gel 1 to the top channel, (b) addition of Gel 2 to top channel and (c) UV crosslinking of the resulting interface between Gel 1 and Gel 2.

**Figure 4.**
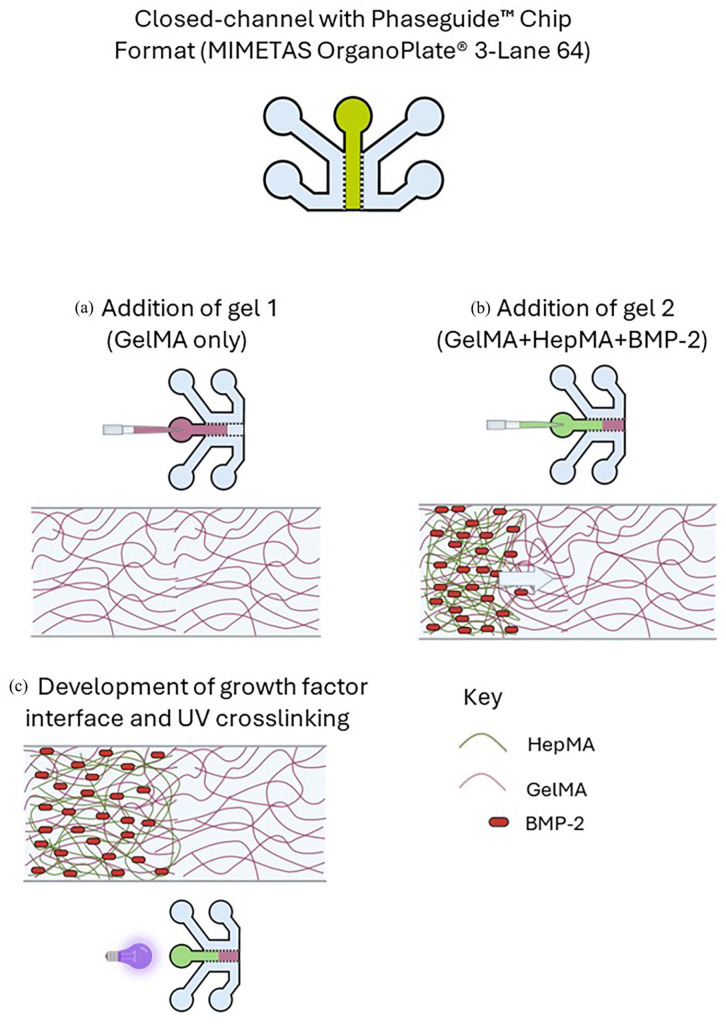
Method to generate spatial distribution of BMP-2 in MIMETAS Organoplate^®^3-lane 64: (a) addition of Gel 1 to the central ECM channel, (b) addition of Gel 2 to the central ECM channel and (c) UV crosslinking of the resulting interface between Gel 1 and Gel 2.

**Figure 5.**
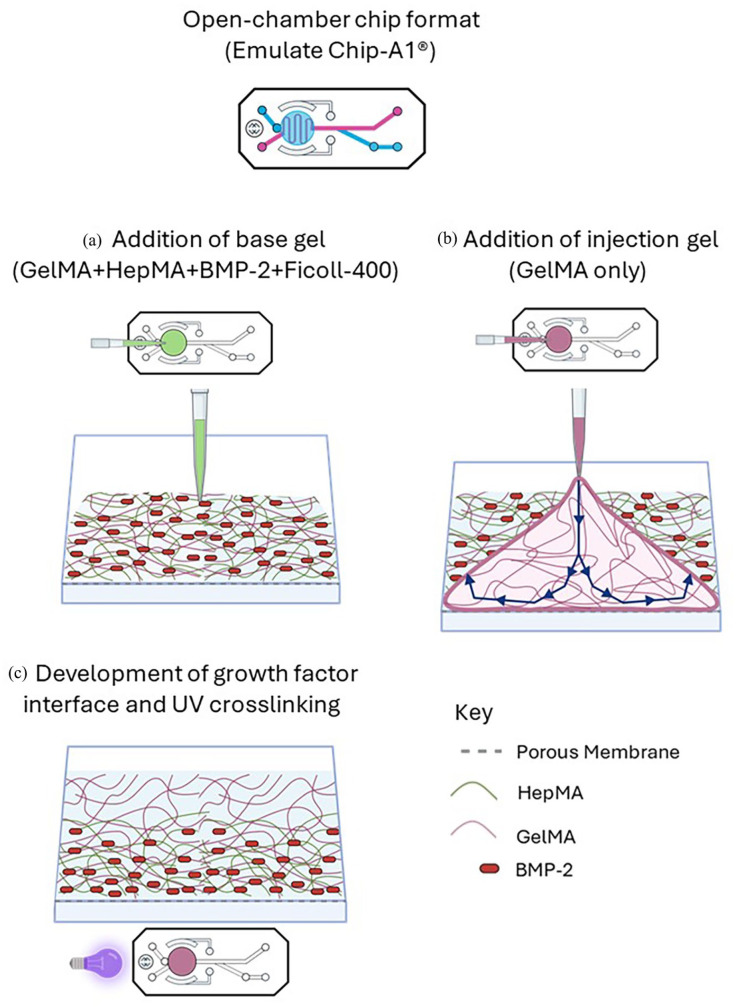
Method to generate spatial distribution of BMP-2 in Emulate Chip-A1^®^: (a) addition of base gel to culture chamber, (b) addition of injection gel to culture chamber and (c) development of the growth factor interface through differential buoyancy, and subsequent UV crosslinking.

1. **Closed-channel with membrane format (Chip-S1**^®^ **):** Spatial patterning of the HepMA-BMP-2 complex in the Chip-S1^®^ was achieved using a displacement method (Figure 3). In brief, the top channel of the chip was filled with Gel 1 (GelMA only) by pipetting 50 µl into the top channel inlet (Figure 3(a)). Gel 2 (GelMA + HepMA + BMP-2) was then immediately pipetted following Gel 1 into the top channel inlet. This created a BMP-2-rich region in the outlet-adjacent half of the channel, and a BMP-2-free region in the inlet-adjacent half, with an interface region between the two (Figure 3(b)). The position of the interface along the length of the top channel was altered by adjusting the volume of Gel 2 pipetted into the channel. The displaced volume of Gel 1 was collected from the surface at the top channel outlet by aspiration. The loaded OOAC were transferred to a UV light box (365 nm, 17 mW/cm^2^) and treated for 3 min (Figure 3(c)). Confirmation of gel curing was carried out by visual inspection. The full length of the chip was imaged by confocal microscopy to assess the distribution of the FITC-BMP-2 along the length of the top channel. The ‘plot profile’ tool in ImageJ (Image J 1.53c, National Institutes of Health, USA) was used to quantify the fluorescence profile along the length of the top channel from the captured images.
**(2) Closed-channel with Phaseguide™ format (MIMETAS OrganoPlate**^®^ **):** Following the same principle as Chip-S1^®^ above, spatial patterning of the HepMA-BMP-2 complex in the OrganoPlate^®^ was also achieved using displacement method (Figure 4). A 0.9 µl drop of Gel 1 (GelMA only) was dispensed on the inlet (Figure 4(a)) of the central channel. As the solution started entering the channel by capillary action, 0.8 µl of Gel 2 (GelMA + HepMA + BMP-2) was then immediately dispensed on the top inlet, creating a BMP-2-rich region in the inlet-adjacent (top half) of the channel, and a BMP-2-free region in the bottom half, with an interface region between the two (Figure 4(b)). The position of the interface along the length of the central channel was altered by adjusting the ratio of Gel 1:Gel 2 (v/v) dispensed on the inlet, never exceeding a combined total volume of 1.7 µl, which resulted in an overflow from the central channel into the adjacent perfusion channel (Supplemental Figure S1). The OOAC were UV cured (Figure 4(c)), imaged using DMi8 Epifluorescence (Leica DMi8 Epifluorescence), combined vertically using image J and a fluorescence profile generated along the length of the central channel.
**(3) Open-chamber format (The Chip-A1**^®^ **):** Spatial patterning of the HepMA-BMP-2 complex in the Chip-A1^®^ was achieved using a differential buoyancy-driven method, specially adapted from Li et al.^1^ to work within the OOAC format (Figure 5). In brief, 80 µl of the base gel (GelMA + HepMA + BMP-2 + Ficoll-400) was pipetted into the accessible chamber (Figure 5(a)) and then 40 µl of the injection gel (GelMA only) was immediately pipetted into the base gel, at an injection rate of ~20 µl/s. (Figure 5(b)) using an E4 Electronic Pipette (Mettler Toldedo, Leicester, UK). Due to the action of buoyancy, the more buoyant gel (the injection gel) rises up within the denser gel (the base gel) creating a gradient between the two, and therefore a gradient of BMP-2. The loaded OOAC were transferred to a UV light box (365 nm, 17 mW/cm^2^) and treated for 3 min (Figure 5(c)). Confirmation of gel curing was carried out by visual inspection. The construct was carefully removed and sectioned using a scalpel to allow for assessment of FITC-BMP-2 gradient by confocal microscopy. Fluorescence intensity profiles were quantified throughout the depth of the gel, as described for Chip-S1^®^.

### Osteochondral development-on-a-chip

In order to demonstrate that the spatial patterning of BMP-2 drives tissue patterning, we established a model of osteochondral development using the closed-channel with membrane format, Chip-S1^®^, as an exemplar application of the methodology.

Human bone marrow mesenchymal stem cells (hBM-MSCs; source: female, caucasian, 72 years old; Promocell, Heidelberg, Germany) were culture-expanded in mesenchymal stem cell growth medium (Promocell) and used in OOAC experiments at passage 5. Gels 1 and 2 were generated as described above, and then individually used to resuspend hBM-MSCs at a density of 3 × 10^6^ cells/ml. For cell-loaded gels, recombinant human BMP-2 (Biotechne, Dublin, Republic of Ireland) was used in the place of FITC-BMP-2. Spatial patterning of the HepMA-BMP-2 complex was performed using the displacement method described above.

**Figure 6.**
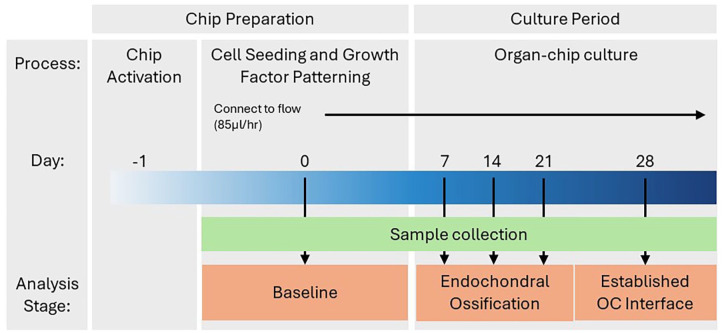
Timeline of cell culture in the Chip-S1^®^. Chips were activated with ER-1 on day −1. Cell-seeded gels were used to develop growth factor patterning within the top channel of the Chip-S1. The organ-chips were cultured for 28 days, with day 0–21 used to examine endochondral ossification and day 28 to study the established osteochondral interface.

Following UV curing, the top channel inlet was sealed using a 1 mm × 1 mm polyethylene terephthalate sticker, while a media droplet was applied to the top channel outlet to prevent drying of the hydrogel. Each chip was connected to a Pod™ and cultured for 28 days (Figure 6) at 5% CO_2_, 37°C in a humidified atmosphere. The bottom channel of the Chip-S1^®^ was continuously perfused, at a rate of 85 µl/hr, with osteochondral differentiation media, comprising high glucose Dulbecco’s modified Eagle medium with pyruvate (ThermoFisher Scientific) supplemented with 1× Insulin-Transferrin-Selenium (ThermoFisher Scientific), 100 × 10^−9^ M dexamethasone, 50 µg/ml l-ascorbic acid, 50 µg/ml l-proline, 2 × 10^−3^ M β-glycerophosphate (all Sigma-Aldrich) and 10 ng/ml TGF-β3 (R&D Systems).

### Endpoint analyses

#### Sample collection

OOAC were harvested at day 0, 7, 14 and 21 to study osteochondral development, and at day 28 to assess the more established osteochondral interface (Figure 6). At each timepoint, OOAC were disconnected from their pods and the bottom channel was gently washed with PBS. Chip-S1^®^ OOAC were carefully halved using a scalpel and separated into +BMP-2 (inlet-side) and −BMP-2 (outlet-side) regions, which were processed separately. The hydrogel in the top channel was digested using proteinase K (3 mg/ml; Merck), which was added to both channels using 200 µl pipette tips inserted into the inlets/outlets. The OOAC halves were incubated for 30 min at 37°C, before the digested hydrogel was carefully collected and centrifuged at 4000×*g*. The resulting cell pellet was lysed in buffer RLT (Qiagen) containing 2% freshly prepared 2 M Dithiothreitol (Sigma-Aldrich), before being stored at −80°C.

#### Cell viability assessment

At day 21, cell distribution was assessed by brightfield microscopy and cell viability was assessed by staining with calcein AM (2.5 µM) and ethidium homodimer-1 (5 µM; both Sigma-Aldrich) in serum-free media, to visualise live and dead cells respectively. Cell viability within stained OOAC was assessed by confocal microscopy.

#### Gene expression analysis

Total RNA was extracted from defrosted cell lysates using the RNeasy^®^ Mini Kit (Qiagen) as per the manufacturer’s instructions. In the final step, RNA was resuspended RNAse-free water (Qiagen). The relative concentration and purity (A260/A280 ratio) of the RNA was quantified using a NanoDrop™ Spectrophotometer (ThermoFisher Scientific). About 100 ng RNA was used to synthesise cDNA by reverse transcription using the QuantiTect Reverse Transcription Kit (Qiagen), according to the manufacturer’s instructions. cDNA was diluted 1:2 in nuclease-free water and qRT-PCR was performed using the TaqMan™ Universal PCR Master Mix (ThermoFisher Scientific) and TaqMan™ primer assays to interrogate a number of genes associated with osteogenic and chondrogenic differentiation (ThermoFisher Scientific; detailed in Supplemental Table 1). Samples were analysed in duplicate. Each reaction consisted of: 1 μl template cDNA, 5 μl PCR Master Mix, 0.5 μl primer assay, and 3.5 μl nuclease-free water (total reaction volume = 10 µl). Samples were loaded in a 384-well plate and thermocycling was performed using the Quantstudio 7 Flex system (Applied Biosystems, Warrington, UK) using the following thermal cycle: hold 2 min at 50°C; hold 10 min at 95°C; 40 cycles: 15 s at 95°C, 1 min at 60°C; hold at 4°C. Cycle threshold (Ct) values were collected at the end of the extension stage of each cycle. Sample gene expression levels were normalised to the geometric mean of their corresponding housekeeping genes.^20^

For the day 0–21 OOAC, the relative expression of each gene at day 7, 14 and 21, compared to that at day 0, was determined using the ΔΔCt method.^21^ For the day 28 OOAC, the relative expression of each gene in the +BMP-2 region, compared to the low BMP-2 region, was determined using the same method. A two-fold change (up- or down-regulated) was deemed biologically significant, and is represented by a dotted line on gene expression figures.

### Human osteochondral tissue collection and processing

In order to generate a comparative gene expression profile for the established osteochondral interface OOAC, samples of mature human cartilage and bone were obtained in compliance with Oxford Musculoskeletal Biobank (OMB15/0177) and MOA003 respectively. For RNA isolation, scaphoid trapezium bone (trapeziectomy) and knee cartilage (total knee arthroplasty) were collected via surgical excision and stored at −80°C in accordance with the national and institutional ethics guidelines, the U.K. Human Tissue Act and the Declaration of Helsinki (12217 and 09/H0606/11).

For gene expression analysis of the human tissues, bone and cartilage samples were snap frozen in liquid nitrogen and pulverised. Tissues, refined to a powder, were added to pre-cooled tubes containing 1 ml TriReagent (Ambion Inc., ThermoFisher Scientific), vortexed for 10 mins and treated with 1-Bromo-3-chloropropane. Post centrifugation, the upper aqueous layer was collected, mixed with ethanol and processed using RNeasy^®^ Mini Kit (Qiagen) as per the manufacturer’s instructions. cDNA synthesis and qRT-PCR, as well as the subsequent data handling, were performed as described above for OOAC.

### Statistical analysis

Statistical analysis was performed using GraphPad Prism version 9.5 for Windows (GraphPad Software, San Diego, California USA). qPCR data was analysed using two-way ANOVA followed by Sidak’s multiple comparisons test or student’s *t*-test, as highlighted in the individual figure legends. All data is presented as mean ± standard deviation (SD) unless otherwise stated. The threshold for statistical significance was set at *p* = 0.05. Statistical significance is displayed as ^#^*p* < 0.05, ^##^*p* < 0.01, ^###^*p* < 0.001 and ^####^*p* < 0.0001.

## Results

### Hydrogel characterisation

**Figure 7.**
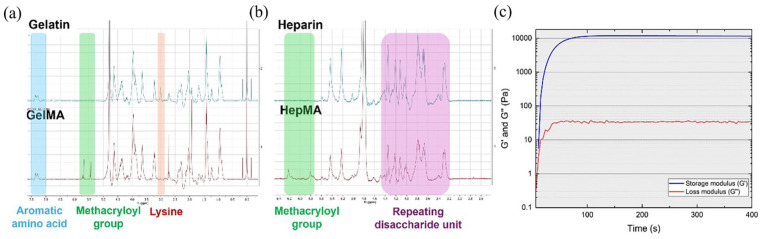
Physico-chemical characterisation of materials: (a) 1H NMR spectra of unmodified gelatin (top) and GelMA (bottom), (b) unmodified heparin (top) and HepMA (bottom) and (c) rheological characterisation of GelMA.

The degree of functionalisation (DoF) of GelMA and HepMA was quantified by 1H NMR spectroscopy. In comparison with the unmodified gelatin spectra (Figure 7(a)), the appearance of the two new peaks at δ = 5.4 and 5.6 ppm (Figure 7(a), green region), corresponding to methacryloyl linkage and the decrease in the free lysine signal at δ = 2.9 ppm (Figure 7(a), red region) confirmed the success of the reaction. The intensity of the aromatic amino acid group (Figure 7(a), blue region) remained constant and was used to normalise the peaks across samples. The DoF, measured by comparing the decrease in the intensity of the lysine signal in GelMA over the unmodified gelatin, was found to be 65%.

Methacryloyl substitution of heparin was found to be 74%, as calculated from the integral of the methacryloyl peaks (Figure 7(b), green region) against the repeating disaccharide unit of heparin (Figure 7(b), purple region), consistent with previous findings.^22^

Rheological measurements of GelMA were conducted to assess its storage (*G*′) and loss (*G*″) moduli during in-situ gelation. Gelation was initiated directly under the rheometer, and a rapid exponential increase in both moduli was observed, marking the onset of gelation. Full gelation occurred around 180 s after UV exposure, with the storage modulus reaching a plateau at 11 kPa, indicating complete crosslinking of the gel (Figure 7(c)).

### Spatial patterning in OOAC

**Figure 8.**
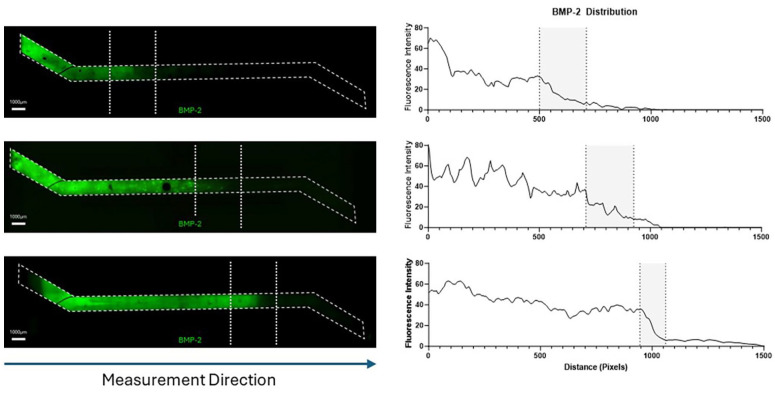
Spatial FITC-BMP-2 distribution in the Chip-S1^®^ (left) with varying volumes (6, 12 and 18 µl: top to bottom) of Gel 2 added to the inlet, and corresponding fluorescence profiles (right). Dotted lines indicated the graded interface between the +BMP-2 and −BMP-2 regions.

**Figure 9.**
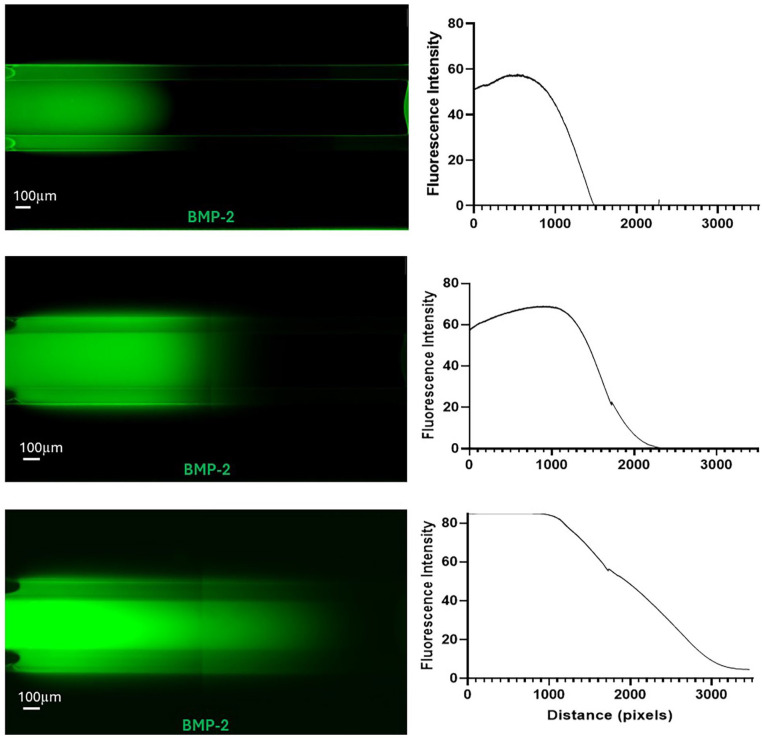
Spatial FITC-BMP-2 distribution in the Organoplate 3-lane 64 (left) with varying volumes of Gel 1 and Gel 2 (respectively, 1.1 µl + 0.6 µl, 0.9 µl + 0.8 µl, 0.6 µl + 1.1 µl; top to bottom) to the central ECM channel, and corresponding fluorescence profiles (right).

**Figure 10.**
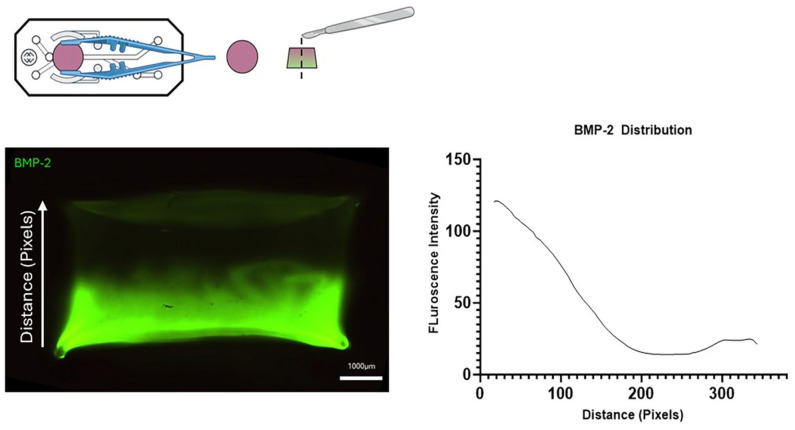
Spatial FITC-BMP-2 distribution in the Chip-A1^®^ (left) and corresponding fluorescence profile (right).

**(1) Closed-channel format (The Chip-S1**^®^ **):** Using the displacement method, we successfully generated a heterogenous distribution of the hepMA-BMP-2 complex along the length of the top channel of the Chip-S1^®^. This was demonstrated using FITC-BMP-2, with confocal imaging revealing the presence of BMP-2 at one end of the channel and its absence at the other (Figure 8). The interface could be moved along the length of the channel by varying the volume of Gel 2 that was pipetted into the top channel in the second stage of the procedure. If 6 µl was used, the interface was located around 1/3 the length of the channel, on the inlet-side (Figure 8, top), 12 µl resulted in a central interface (Figure 8, middle) and 18 µl resulted in an interface located around 2/3 the length of the channel, on the outlet-side (Figure 8, bottom). There was a graded interface between the +BMP-2 and −BMP-2 regions in all cases, as highlighted by the corresponding fluorescence profiles. It was noted that upon UV curing, the hydrogel retracted in the top channel of the Chip-S1^®^, leaving a consistent gap between the hydrogel and the walls of the OOAC.
**(2) Closed-channel with Phaseguide™ format (MIMETAS OrganoPlate**^®^ **):** Using a similar displacement method, spatial distribution of the hepMA-BMP-2 complex could be varied along the length of the middle channel in OrganoPlate^®^ (Figure 9). First, by dispensing different volumes of Gel 2 in the central ECM channel, an optimal loading volume of 1.7 µl was established for GelMA hydrogel (Supplemental Figure 1). Then the interface was moved along the length of the channel by varying the ratios of Gel1: Gel 2 (v/v) that were dispensed on the inlet, never exceeding the optimised volume. Using 1.1 µl and 0.6 µl of the Gel 1 and Gel 2 respectively, the interface was located around 1/3 of the length of the inlet-adjacent half (Figure 9, top), with 0.9 µl (Gel 1) and 0.8 µl (Gel 2) positioning the interface in the centre of the channel (Figure 9, middle) and 0.6 µl (Gel 1) and 1.1 µl (Gel 2) resulting in an interface around 2/3 the length of the channel, on the far side (Figure 9, bottom). There was a graded interface between the +BMP-2 and −BMP-2 regions in all cases, as highlighted by the corresponding fluorescence profiles.
**(3) Open-chamber format (The Chip-A1**^®^ **):** Using the differential buoyancy method, we successfully generated a heterogenous distribution of BMP-2 in the top culture chamber of the Chip-A1^®^. This was demonstrated using FITC-BMP-2, with confocal imaging revealing the presence of high-BMP-2 at the bottom, and low-BMP-2 at the top of the sectioned construct (Figure 10). There was a graded interface between the high- and low-BMP-2 regions, as highlighted by the corresponding fluorescence profile. It was noted that upon UV curing, the hydrogel retracted in the culture chamber of the Chip-A1, leaving a gap between the hydrogel and the walls of the OOAC.

### Cell viability and distribution

**Figure 11.**
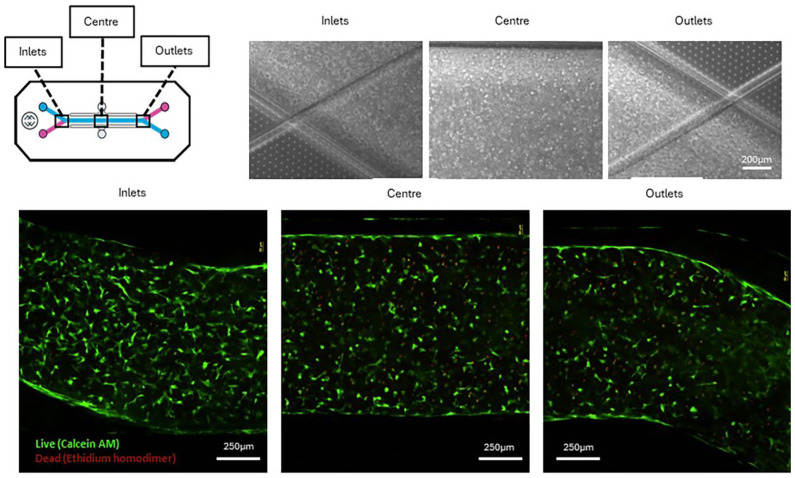
Top: cell distribution in the inlets, centre and outlets of the top channel of the chip seeded with the hBM-MSC-loaded hydrogels at day 21. Bottom: cell viability assessed by live/dead staining in the same regions at day 21.

Brightfield imaging revealed consistent cell distribution in the inlet, centre and outlet regions of the top channel of the OOAC after 21 days of culture (Figure 11). Also evident in the brightfield images is the slight retraction of the hydrogel from the walls of the top channel. Live/dead staining, with calcein AM/ethidium homodimer-1 respectively, revealed excellent cell viability in the gel-loaded top channel, in the three different regions, after 21 days of culture (Figure 11).

### Gene expression in the developing osteochondral interface

**Figure 12.**
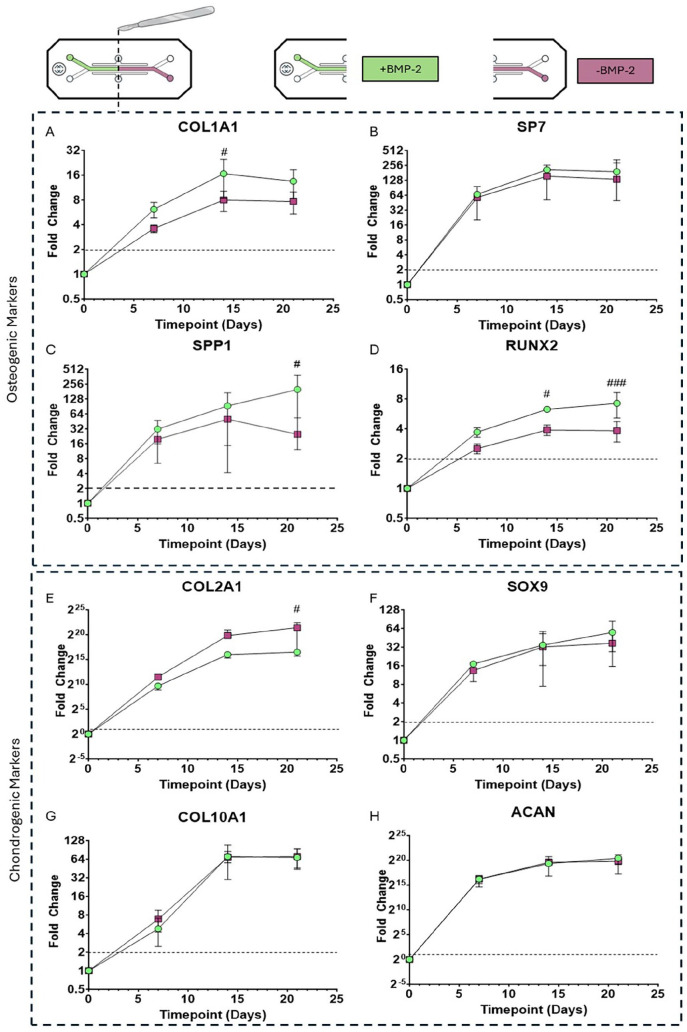
Gene expression profiles of osteogenic (A-D) and chondrogenic (E-H) markers in assessment of endochondral ossification in the organ-chip. *N* = 3 chips per timepoint. # denotes significant difference between +BMP-2 and −BMP-2 region at a specific timepoint. Dotted lines denote a two-fold upregulation.

At days 0, 7, 14 and 21 OOAC (*n* = 3) were harvested and divided into +BMP-2 and −BMP-2 regions for gene expression analysis. The gene expression of the +BMP-2 and −BMP-2 regions at the four different timepoints were normalised to the day 0 expression. There was a general increase in all of the genes assessed from day 0 to day 7, and from day 7 to day 14, in both the +BMP-2 and −BMP-2 regions, before several of the genes plateaued, or even decreased in expression between day 14 and day 21 (Figure 12). Comparative gene expression analysis revealed distinct expression profiles between the +BMP-2 and −BMP-2 regions in several of the genes interrogated. In general, the expression of osteogenic markers was more sustained, and the expression of chondrogenic markers lower, in the +BMP2 region of the OOAC. At day 14, the expression of COL1A1 was significantly higher in the +BMP-2 region compared to the −BMP-2 region (16.66 ± 8.28 vs 7.99 ± 2.20 respectively; *p* = 0.0396; Figure 12(a)), as was the expression of SPP1 (198.67 ± 186.54 vs 24.78 ± 28.26; *p* = 0.0441; Figure 12(c)) and RUNX2: 6.28 ± 0.054 vs 3.89 ± 0.48; *p* = 0.0115; Figure 12(d)). At day 21 there was still a significant difference in RUNX2 expression, with a higher expression in the +BMP-2 region compared to the −BMP-2 region (7.21 ± 2.070 vs 3.81 ± 0.90; *p* = 0.0005; Figure 12(d)). There was also a significant difference in the expression of COL2A1, a marker of chondrogenic differentiation, at day 21, with significantly higher expression in the −BMP-2 region, compared to the +BMP-2 region (92058.57 ± 8996.67 vs 2790606 ± 2737369 respectively; *p* = 0.0237; Figure 12(e)).

### Gene expression in the established osteochondral interface

**Figure 13.**
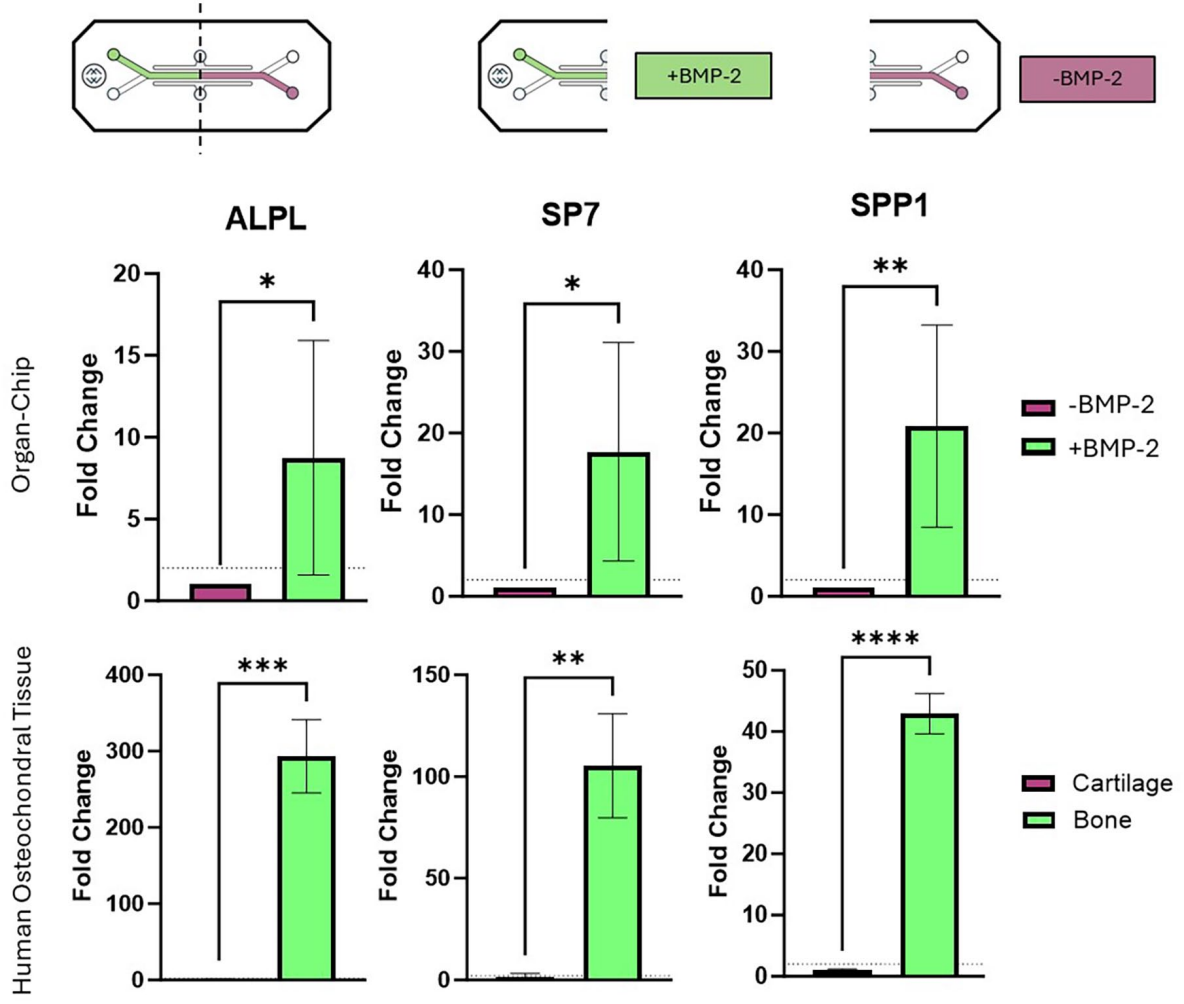
Comparative gene expression profiles of osteogenic markers in the established osteochondral interface chip at day 28. *n* = 6 top. *N* = 3 bottom. *denotes significant difference between +BMP-2 and −BMP2 region (top) or between bone and cartilage (bottom) for each gene at day 28. Dotted lines denote a twofold upregulated.

At day 28, OOAC (*n* = 6) were harvested and divided into +BMP-2 and −BMP-2 regions for the analysis of genes indicative of osteogenic differentiation (ALPL, SP7 and SPP1). The gene expression of the +BMP-2 and −BMP-2 regions were normalised to the expression in the −BMP-2 region. All of the genes assessed were more highly expressed in the +BMP-2 region, with a significant fold change in ALPL (8.752 ± 7.162; *p* = 0.0432), SP7 (17.7 ± 13.38, *p* = 0.025) and SPP1 (20.83 ± 12.37, *p* = 0.009; Figure 13). The expression of the same genes was also assessed in the same manner in mature, human cartilage and bone and a similar expression profile was highlighted. All of the genes assessed were more highly expressed in the bone compared to the cartilage, with a significant fold change in ALPL (293 ± 48.14; *p* = 0.0005), SP7 (105.4 ± 25.53; *p* = 0.0022) and SPP1 (42.93 ± 3.284; *p* < 0.0001; Figure 13).

## Discussion

While organ-on-a-chip offers a range of benefits over traditional in vitro models, methods to generate morphogen-driven spatiotemporal tissue patterning in these systems have yet to be developed. Here, the authors sought to address this technical deficit, by generating and optimising such methods, and by ensuring that they could be utilised across a broad range of OOAC systems. The subsequent aim was to demonstrate the efficacy of these techniques by modelling the development of the osteochondral interface, by endochondral ossification, as an exemplar application.

The methods developed in the present study exploited the capacity of the polyanionic glycosaminoglycan (GAG), heparin, to act as a macromolecular host through its ability to sequester cationic proteins, such as growth factors.^23^ The addition of methacryloylate groups allows for the HepMA to be covalently crosslinked to both itself, and to the surrounding bulk hydrogel, GelMA.^1^ In combination, these properties allow for the spatial patterning of sequestered growth factors via the spatial patterning of HepMA within the GelMA; patterning which can then be immobilised by the addition of a photoinitiator and the application of UV light. Here, the authors demonstrated the effectiveness of this methodology in OOAC systems by successfully generating spatial patterning of the morphogen, BMP-2, in systems representative of three OOAC formats: (1) closed-channel with membrane (the Chip-S1^®^), (2) closed-channel with Phaseguides™ (MIMETAS Organoplate^®^) and (3) open-chamber OOAC (the Chip-A1^®^).

Due to the configurational dissimilarities of the three OOAC systems, different approaches were required to generate spatial patterning in each. In the Chip-S1^®^ and Organoplate^®^ a displacement method was utilised, by which one hydrogel containing the HepMA-BMP-2 complex was used to displace a controlled volume of another without HepMA-BMP-2. This was made possible by the unique behaviour of liquids in microfluidic channels, by which the displacement of one by another, even those which are miscible, is almost complete, with mixing at the interface due to diffusion alone. This phenomenon was evident in the FITC-BMP-2 experiments in the Chip-S1^®^ and Organoplate^®^, with a graded, rather than abrupt, interface generated between regions of +BMP-2 and −BMP-2. Of particular note was the fact that the displacement method was successful in a system such as the Organoplate^®^, which has Phaseguides™ and therefore is heavily reliant on surface tension to prevent leakage into adjacent channels. This system also had the added benefit of control over the location of the interface by altering the displacement volume, a feature which could be exploited in future studies to create tissue gradients. The arrangement of the open-chamber Chip-A1^®^ precluded the use of a displacement method but allowed for the use of a buoyancy-driven method. This method was first described by Li and colleagues for the development of gradient hydrogels out of OOAC systems,^1^ and it could be readily adapted to the open-chamber OOAC system. Again, this was demonstrated using FITC-BMP-2, and a transitional, top-to-bottom gradient of BMP-2 was evident in the hydrogel removed from the culture chamber of the Chip-A1^®^.

The majority of academic and commercially available OOAC systems can be stratified into one of the formats discussed, comprising closed channels (with either membranes, pillars or Phaseguides™), open chambers or a combination of these features. Therefore, the methods described in the present work could be applied to a wide range of OOAC models, further augmenting their utility. In addition, heparin is able to sequester a wide range of proteins, including growth factors, such as fibroblast growth factor, neural growth factor and TGF-β,^24^ cytokines, such as interferon gamma and interleukin-6^25^ and chemokines such as CXCL11, CCL21.^26,27^ Additionally, other negatively charged GAGs, such as chondroitin sulphate, can also be used to sequester proteins and could therefore act as an alternative macromolecular host for such morphogens.^28^ Thus, the use of the GAG-protein patterning methodology, within OOAC, could be used for an extensive range of applications.

The authors subsequently demonstrated that the HepMA-BMP-2 patterning in the Chip-S1^®^ was functionally relevant and could be used within the Chip-S1^®^ to emulate OC development. In vivo, OC development is a complex and tightly controlled process under the command of a range of morphogens, including BMP-2 and TGF-β3, and broadly occurs in postnatal development in two stages in a region called the growth plate.^16,29^ The first stage involves the condensation and differentiation of mesenchymal stem cells into chondrocytes, partly mediated again by TGF-β3.^30^ The chondrocytes secrete ECM to form a cartilage template; a process known as chondrogenesis.^31^ In the second stage, the chondrocytes become hypertrophic, and the cartilage template is gradually remodelled into bone by osteoblasts, which arise from both invasion and local MSC differentiation, in a process termed endochondral ossification.^31,32^ BMP-2, as one of the most potent inducers of osteogenic differentiation, plays a crucial role in this process.^29^

**Figure 14.**
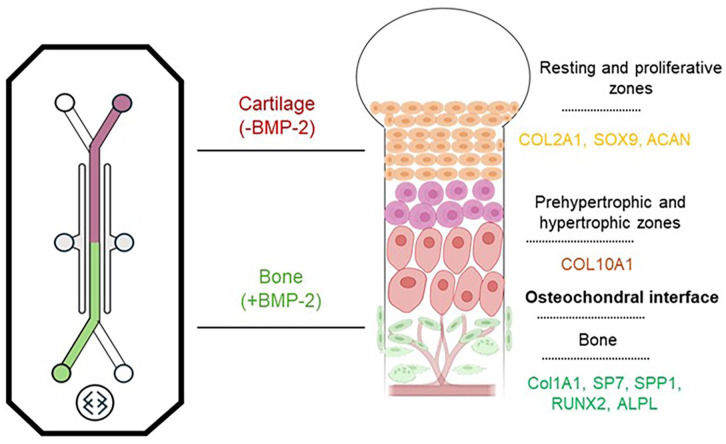
Human growth plate schematic. Characteristic gene expression profile across the human growth plate which was replicated in the matched regions of the osteochondral development-on-a-chip.

The process of osteochondral development was recapitulated in the OOAC system, using hBM-MSCs and spatial patterning of the HepMA-BMP-2 complex as described for the Chip-S1^®^. Figure 14 presents an overview of the characteristic gene expression in the different regions of the growth plate that we were able to recapitulate in the OOAC. Gene expression analysis revealed an increase in the expression of key chondrogenic markers (COL2A1, SOX9, COL10A1 and ACAN) in hBM-MSCs in both the low and +BMP-2 regions up to day 21, reflective of the chondrogenesis stage of OC development throughout the top channel of the OOAC. In vivo, this stage is largely regulated by TGF-β3. As described, the bottom channel of the OOAC was continuously perfused with a mixed osteochondral media, developed by Li et al.^1^ Critically, this media contained TGF-β3 which, mirroring results by these authors in off-chip hydrogels, stimulated tissue-wide chondrogenesis in the hydrogel in the top channel of the Chip-S1^®^. Li et al.^1^ also showed that BMP-2 was gradually released from HepMA over the course of 28 days. This was again reflected in the osteochondral development chip, with a more localised osteogenic response, that drove the divergence in gene expression from day 14 of key osteogenic markers (COL1A1, RUNX2, SP7, SPP1) and of COL2A1 between the +BMP-2 and −BMP-2 regions. This spatial variation in gene expression emulated that seen across the human growth plate, the site of endochondral ossification. Interestingly, the expression of COL10A1, which is indicative of the central, hypertrophic chondrocyte region of the growth plate, was almost identical in both the +BMP-2 and −BMP-2 regions of the OOAC. This is possibly due to the commitment of the hBM-MSCs, throughout the OOAC, to ossification via the endochondral pathway, and is therefore representative of the second stage of this process in which the chondrocytes become hypertrophic.^18,31^ A similar phenomenon has been previously observed in cartilage organoids, engineered using hBM-MSCs, in which COL10A1 gene expression and collagen type X protein deposition were observed even after 14 days of culture in chondrogenic differentiation media, which similarly contained TGF-β3.^33^ A technical limitation may also have contributed to a reduction in the spatial resolution in zonal gene expression in comparison to the native growth plate. OOAC were halved for gene expression analysis, meaning that the central, hypertrophic zone was likely to have been divided between the two halves. Ideally, the OOAC would be divided into three regions for gene expression analysis, but the low channel volume and therefore low cell number in the Chip-S1^®^, would limit the RNA available for reverse transcription.

As well as studying endochondral ossification in the OOAC from day 0 to 21, the authors were keen to interrogate the more established osteochondral interface in the OOAC, and to compare the expression of selected genes to mature human cartilage and bone. Li and colleagues previously demonstrated clear distinction between bone and cartilage elements in their model at day 28, which was therefore considered as the endpoint in the osteochondral OOAC.^1^ The profile of expression of the key osteogenic genes ALPL, SP7 and SPP1 in mature cartilage and bone was mirrored in the osteochondral OOAC. Specifically, all three genes showed significant fold changes between the bone- (+BMP-2) and cartilage-like (−BMP-2) region of the osteochondral OOAC and between mature human bone and cartilage. These fold changes were also of similar magnitude, again highlighting the ability of the OOAC to emulate human tissue differences. Thus, the authors successfully demonstrated that the methodologies to spatially pattern BMP-2 in the OOAC could be used to study both endochondral ossification and the established osteochondral interface. Work is ongoing within our group to adapt and exploit the methodologies described for alternative materials, alternative OOAC and alternative applications. While beyond the scope of the present, methodologically-focussed work, further comparative biological characterisation of the −BMP-2 and +BMP-2 regions, as well as the interface between the two, would be of great interest. Methods for the immunohistological assessment of key tissue markers within the OOAC are being optimised for future work.

## Conclusion

The authors successfully developed methods to spatially pattern hydrogel-embedded bioactive signalling molecules, within three different types of OOAC, namely: the closed-channel with membrane Emulate Chip-S1^®^, the closed-channel with Phaseguides™ MIMETAS OrganoPlate ^®^, and the open-chamber Emulate Chip-A1^®^ We then use the Emulate Chip-S1^®^ to show how this growth factor patterning drives spatiotemporal patterning of human tissues within the OOAC. With the process of endochondral ossification as an exemplar application of these methodologies, we demonstrate the ability to replicate phenotypic features of both the developing and established osteochondral interface. However, these methods are not limited to this single model, nor to the platforms described, and can applied across a wide range of OOAC platforms, materials and tissues. These spatial patterning methodologies will therefore support the development of next generation OOAC models, incorporating tissue heterogeneity to enhance predictive power for fundamental research and therapeutics testing.

## Supplemental Material

sj-jpg-1-tej-10.1177_20417314251326256 – Supplemental material for Engineering growth factor gradients to drive spatiotemporal tissue patterning in organ-on-a-chip systems Supplemental material, sj-jpg-1-tej-10.1177_20417314251326256 for Engineering growth factor gradients to drive spatiotemporal tissue patterning in organ-on-a-chip systems by Timothy Hopkins, Swati Midha, Simon Grossemy, Hazel R. C. Screen, Angus K. T. Wann and Martin M. Knight in Journal of Tissue Engineering

sj-jpg-2-tej-10.1177_20417314251326256 – Supplemental material for Engineering growth factor gradients to drive spatiotemporal tissue patterning in organ-on-a-chip systems Supplemental material, sj-jpg-2-tej-10.1177_20417314251326256 for Engineering growth factor gradients to drive spatiotemporal tissue patterning in organ-on-a-chip systems by Timothy Hopkins, Swati Midha, Simon Grossemy, Hazel R. C. Screen, Angus K. T. Wann and Martin M. Knight in Journal of Tissue Engineering

## Statements and Declarations

**Ethical considerations:** Samples of mature human cartilage and bone were obtained in compliance with Oxford Musculoskeletal Biobank (OMB15/0177) and MOA003 respectively. Scaphoid trapezium bone (trapeziectomy) and knee cartilage (total knee arthroplasty) were collected via surgical excision and stored at −80°C in accordance with the national and institutional ethics guidelines, the U.K. Human Tissue Act and the Declaration of Helsinki (12217 and 09/H0606/11).

**Consent to participate:** Written informed consent for the collection of the above tissues was obtained by the Oxford Musculoskeletal Biobank in line with national and international ethical standards.

**Consent for publication:** Not applicable.

**Data availability:** All data that support the findings of this study are included within the article (and any Supplemental Files). Any additional information will be provided on request directed to the corresponding author (tim.hopkins@qmul.ac.uk).
