# Supplementary figures and images for: Engineering growth factor gradients to drive spatiotemporal tissue patterning in organ-on-a-chip systems
Source: J Tissue Eng. 2025 Apr 18;16:20417314251326256. doi: 10.1177/20417314251326256 (PMC12033634; doi:10.1177/20417314251326256)

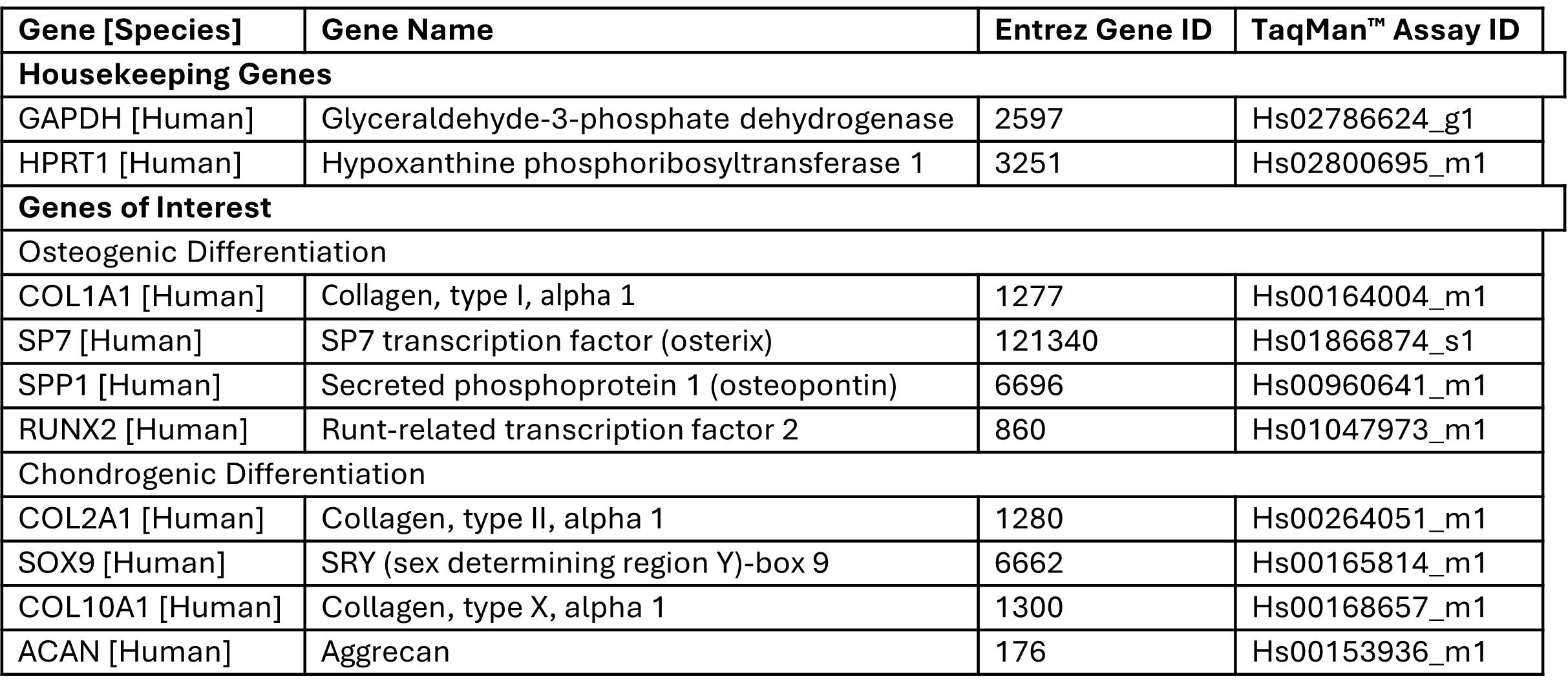

Supplement: sj-jpg-1-tej-10.1177_20417314251326256 – Supplemental material for Engineering growth factor gradients to drive spatiotemporal tissue patterning in organ-on-a-chip systems [file sj-jpg-1-tej-10.1177_20417314251326256.jpg]

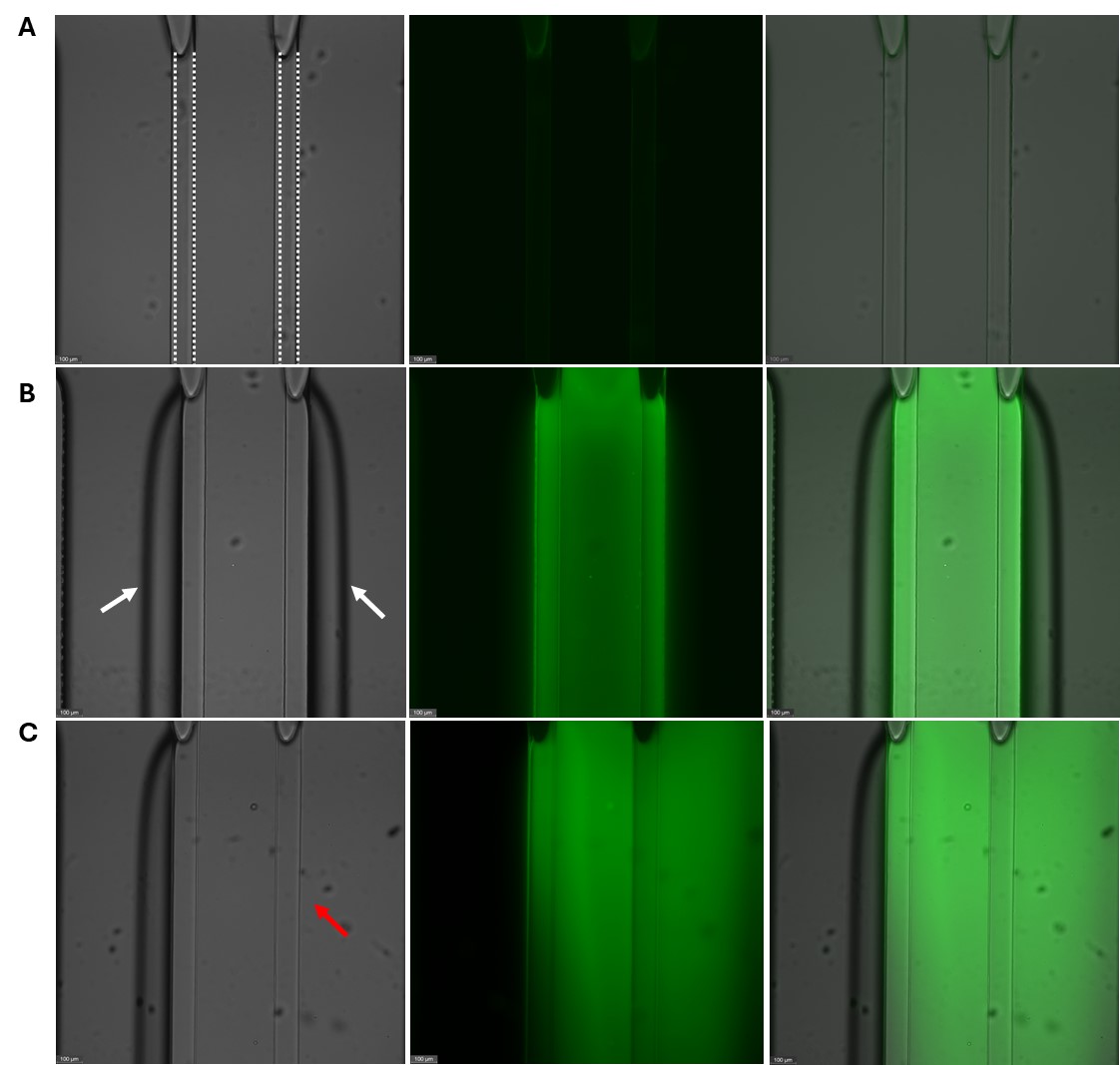

Supplement: sj-jpg-2-tej-10.1177_20417314251326256 – Supplemental material for Engineering growth factor gradients to drive spatiotemporal tissue patterning in organ-on-a-chip systems [file sj-jpg-2-tej-10.1177_20417314251326256.jpg]
